# Phenotypic Characterization of miR-92a^−/−^ Mice Reveals an Important Function of miR-92a in Skeletal Development

**DOI:** 10.1371/journal.pone.0101153

**Published:** 2014-06-30

**Authors:** Daniela Penzkofer, Angelika Bonauer, Ariane Fischer, Alexander Tups, Ralf P. Brandes, Andreas M. Zeiher, Stefanie Dimmeler

**Affiliations:** 1 Institute for Cardiovascular Regeneration, Centre of Molecular Medicine, J. W. Goethe University Frankfurt, Frankfurt am Main, Germany; 2 Department of Animal Physiology, Faculty of Biology, Philipps University Marburg, Marburg, Germany; 3 Institute for Cardiovascular Physiology, J. W. Goethe University Frankfurt, Frankfurt am Main, Germany; 4 Department of Cardiology, Internal Medicine III, J. W. Goethe University Hospital Frankfurt, Frankfurt am Main, Germany; 5 German Centre of Cardiovascular Research (DZHK), Partner Site RheinMain, Frankfurt am Main, Germany; Harvard Medical School, United States of America

## Abstract

MicroRNAs (miRNAs, miRs) emerged as key regulators of gene expression. Germline hemizygous deletion of the gene that encodes the miR-17∼92 miRNA cluster was associated with microcephaly, short stature and digital abnormalities in humans. Mice deficient for the miR-17∼92 cluster phenocopy several features such as growth and skeletal development defects and exhibit impaired B cell development. However, the individual contribution of miR-17∼92 cluster members to this phenotype is unknown. Here we show that germline deletion of miR-92a in mice is not affecting heart development and does not reduce circulating or bone marrow-derived hematopoietic cells, but induces skeletal defects. MiR-92a^−/−^ mice are born at a reduced Mendelian ratio, but surviving mice are viable and fertile. However, body weight of miR-92a^−/−^ mice was reduced during embryonic and postnatal development and adulthood. A significantly reduced body and skull length was observed in miR-92a^−/−^ mice compared to wild type littermates. µCT analysis revealed that the length of the 5^th^ mesophalanx to 5^th^ metacarpal bone of the forelimbs was significantly reduced, but bones of the hindlimbs were not altered. Bone density was not affected. These findings demonstrate that deletion of miR-92a is sufficient to induce a developmental skeletal defect.

## Introduction

MicroRNAs (miRNAs, miRs) are key regulators of gene expression by binding to target mRNAs thereby inducing RNA degradation or blocking translation. Despite ample evidence that miRNAs modulate pathophysiological processes [1]–[3], only few miRNAs have been associated with developmental defects. The polycistronic miR-17∼92 cluster, which comprises the mature miRNAs miR-17, -18a, -19a/b, -20a, and miR-92a, contributes to the pathogenesis of a variety of human diseases, including cancer, cardiovascular disease and congenital developmental defects [2], [4], [5]. Deletion of the miR-17∼92 cluster resulted in defects of heart and lung development, and homozygote mice postnatally died [6]. Particularly B cell development was significantly impaired [6]. A germline hemizygous deletions of MIR17HG, encoding the miR-17∼92 polycistronic miRNA cluster, was observed in patients with Feingold syndrome [7], which is an autosomal dominant syndrome whose core features are microcephaly, relative short stature and digital anomalies, particularly brachymesophalangy of the second and fifth fingers and brachysyndactyly of the toes [8]. Several key features of this phenotype were mimicked in mice harboring targeted deletion of the miR-17∼92 cluster [7]. MiR-17-92^Δ/+^ mice showed a reduced body mass, skull size and a reduced length of the 5^th^ mesophalanx to 5^th^ metacarpal bone [7].

Consistently with the up-regulation of the miR-17∼92 cluster in tumors, miR-92a is highly expressed in colon cancer tissues and targets the anti-apoptotic molecule BCL-2-interacting mediator of cell death (BIM) and the tumor suppressor PTEN [9], [10]. In the cardiovascular system, inhibition of miR-92a enhances neovascularization after hind limb or myocardial ischemia [11] and a pro-angiogenic effect after miR-92a inhibition contributed to fracture healing [12]. MiR-92a was shown to be down-regulated in endothelial cells by shear stress and its inhibition increased the expression of anti-atherosclerotic factors such as Krüpple-like factor 2 (KLF2) and the deacetylase SIRT1 leading to an improved vascular healing and inhibition of atherosclerosis [13]–[16]. However, the contribution of miR-92a for the observed defects in miR-17∼92 cluster deficient mice has not been elucidated.

Here, we demonstrate that genetic deletion of miR-92a does not affect heart and lung development or B cell survival, but reflects the skeletal development defects observed in full cluster knock-outs.

## Methods

### Generation of constitutive and conditional miR-92a deficient mice

The study was performed according to national guidelines and was approved by the ethical committee (Regierungspräsidium Darmstadt). The constitutive and conditional deletion of miR-92a-1, which is expressed by the miR-17∼92 cluster, was generated by homologous recombination in 129Sv/Pas embryonic stem (ES) cells by genOway (Lyon, France). For this purpose, a targeting vector containing the homologous genomic miR-92a-1 sequences flanked by loxP sites and a neomycin gene flanked by FRT sites was used. For the generation of constitutive miR-92a deficient mice (miR-92a^−/−^), the miR-92a recombined chimeric mice were bred with a deleter line constitutively expressing the Cre recombinase. For the generation of conditional endothelial, cardiomyocyte- or hematopoietic-miR-92a knock-out mice (miR-92a^fl/fl^Tie2-Cre, miR-92^fl/fl^αMHC-Cre and miR-92^fl/fl^Vav-Cre), the miR-92a recombined chimeric mice were first bred with C57BL/6J wild type and Flp recombinase expressing deleter mice to excise the neomycin selection cassette and then mated with the respective Cre deleter lines expressing Cre recombinase under the control of Tie2, αMHC or Vav promoter.

### Mouse genotyping

DNA isolation with either REDExtract-N-Amp Tissue PCR Kit (Sigma Aldrich, St. Louis, MO, USA) or DNeasy Blood & Tissue Kit (Qiagen, Hilden, Germany) was done according to the manual. Following primers (5′–3′) were used for genotyping PCRs. Cpxm1 was used as positive control for Cre-PCR.

miR-92a KO forw: CTGTCCTGTTATTGAGCACTGGTCTATGG.

miR-92a KO rev: AAGACATTAGTAACCCACCCCCATTCC.

miR-92a flox forw: AATGTGTGTCTTAGAGGCCTAGTAGTGAAGAGG.

miR-92a flox rev: CACCCCCATTCCTGAAAGCTTATAGC.

Cre forw: CCATCTGCCACCAGCCAG.

Cre rev: TCGCCATCTTCCAGCAGG.

Cpxm1 forw: ACTGGGATCTTCGAACTCTTTGGAC.

Cpxm1 rev: GATGTTGGGGCACTGCTCATTCACC.

Vav-Cre forw: CTCTGACAGATGCCAGGACA.

Vav-Cre rev: ACACCATTCTTTCTGACCCG.

### RNA isolation and determination of miRNA expression levels

Animal tissues were lysed in Qiazol (Qiagen, Hilden, Germany) and homogenized using FastPrep-24 instrument (MP Biomedicals, Solon, OH, USA). Total RNA including miRNAs was isolated using miRNeasy Mini Kit (Qiagen, Hilden, Germany) according to the manufacturer’s protocol.

For determination of miRNA expression levels, TaqMan MicroRNA Assays (Applied Biosystems, Foster City, CA, USA) were used. qPCRs were done on a StepOnePlus device (Applied Biosystems, Foster City, CA, USA). MiRNA expression data were normalized to U6 as endogenous control. Relative expression levels were calculated as 2^−ΔCT^.

### Blood pressure and heart rate measurement

Blood pressure and heart rate were measured by tail-cuff method using a BP-2000 Blood Pressure Analysis System (Visitech Systems, Apex, NC, USA). Animals were adapted to the experimental set-up on 5 consecutive days. On each of the following 5 days, blood pressure and pulse were measured in at least 2 sets of 10 measurements. For determination of blood pressure and heart rate, at least 17 successful measurements on each of the 5 consecutive days were used for final quantification of blood pressure and heart rate.

### Blood count

Animals were sacrificed by cervical dislocation under isoflurane anesthesia and blood was obtained from the Vena cava inferior supplemented with 20 µl EDTA (10 µg/µl). Following blood parameters were measured or calculated using either *scil Vet abc* Plus + (scil animal care company GmbH, Viernhein, Germany) or VetScan HM5 (Abaxis Inc., Union City, CA, USA): White blood cells (WBC), red blood cells (RBC), platelets (PLT), hemoglobin (HGB), hematocrit (HCT), mean corpuscular hemoglobin (MCH), mean corpuscular volume (MCV), mean corpuscular hemoglobin concentration (MCHC).

### Micro-computed tomography (µCT)

A SkyScan 1176 micro-CT system (RJL Micro & Analytic GmbH, Karlsdorf-Neuthard, Germany) was used to perform oversize scans of WT and miR-92a^−/−^ mice with the following settings: 180°C scan; rotation step = 2.5 deg.; averaging = 10 frames; image pixel size = 35 µm; filter = Al 0.2 mm. Mice were sacrificed by cervical dislocation under isoflurane anesthesia before scanning.

### Dual energy X-ray absorptiometry (DEXA)

Bone density measured by dual energy X-ray absorptiometry (DEXA) was performed using a Lunar PIXImus Mouse Densitometer (GE *Lunar*, Madison, WI, USA) under isoflurane anesthesia.

### Heart and muscle tissue sections, hematoxylin-eosin and lectin staining

Hearts were fixed with 4% formalin, embedded in paraffin, cut into 4 µm thick longitudinal sections and stained with hematoxylin-eosin. 10 µm frozen skeletal muscle sections of the lower leg of the hind limb were used to stain myofibril membranes using anti-laminin (Abcam) followed by anti−rabbit-Alexa 647 (Invitrogen) and capillaries with biotinylated isolectin B4 (Vector) followed by SAV-Alexa 488 (Invitrogen).

### Fluorescence activated cell sorting (FACS) of blood and bone marrow

After cervical dislocation under isoflurane anesthesia, blood was obtained from the vena cava inferior supplemented with 200 µg EDTA and 2–4% BSA. Bone marrow was isolated from femurs of both legs of each animal by using PBS with 2–4% BSA. Bone marrow cells were pelleted by centrifugation at 500×g and 4°C for 10 min. Pelleted cells were resuspended in PBS with 2–4% BSA.

Blood and bone marrow were incubated with anti-B220-Pac Blue (BD, Franklin Lakes, NJ, USA); anti-CD19-APC (Miltenyi Biotec GmbH, Bergisch Gladbach), anti-IgM-FITC (BD, Franklin Lakes, NJ, USA), anti-CD43-PE (BD, Franklin Lakes, NJ, USA) and anti-CD45-perCP (BD, Franklin Lakes, NJ, USA) at 4°C for 30 min. Erythrocytes were lysed by using 2 ml FACS lysing solution (BD, Franklin Lakes, NJ, USA) for 4 min (bone marrow) or 8 min (blood). After centrifugation at 800×g and 4°C for 10 min pelleted cells were resuspended in PBS and again centrifuged at 800×g and 4°C for 10 min. Afterwards cells were fixed with 4% formalin and measured by FACS.

### Statistical analysis

For data illustration and statistical analysis, GraphPad Prism version 5.03 (GraphPad Software, San Diego, CA, USA) was used. Data are expressed as mean ± standard error (SEM).

Two treatment groups were compared by students t-test. If not indicated in the figure legend, three treatment groups were compared by one-way analysis of variance (ANOVA) followed by Bonferroni post-hoc analysis. Results were considered statistically significant when *P*<0.05.

The observed distribution of genotypes from offspring was compared to expected Mendelian ratios by chi-square test. Results were considered statistically significant when *P*<0.05. If chi-square test was statistically significant, following further analysis were performed: To test whether the number of observations in each group, say k among n trials, could still plausibly explained by the assumed probabilities, 95%-confidence intervals [p_1_, p_2_] for binomial proportions have been calculated, using the Software R. The edges, defined by P_p1_(X≥k) = 0.025 and P_p2_(X≤k) = 0.025, are computable as quantiles for Beta distributions, according to Clopper-Pearson: p_1_ = F^−1^(0.025; k, n−k+1) and p_2_ = F^−1^(0.025; k+1, n−k); where F(q; a, b) denotes the distribution function for a Beta distribution with shape parameters a, b.

## Results

### Expression of miR-92a and other cluster members

**Figure 1:**
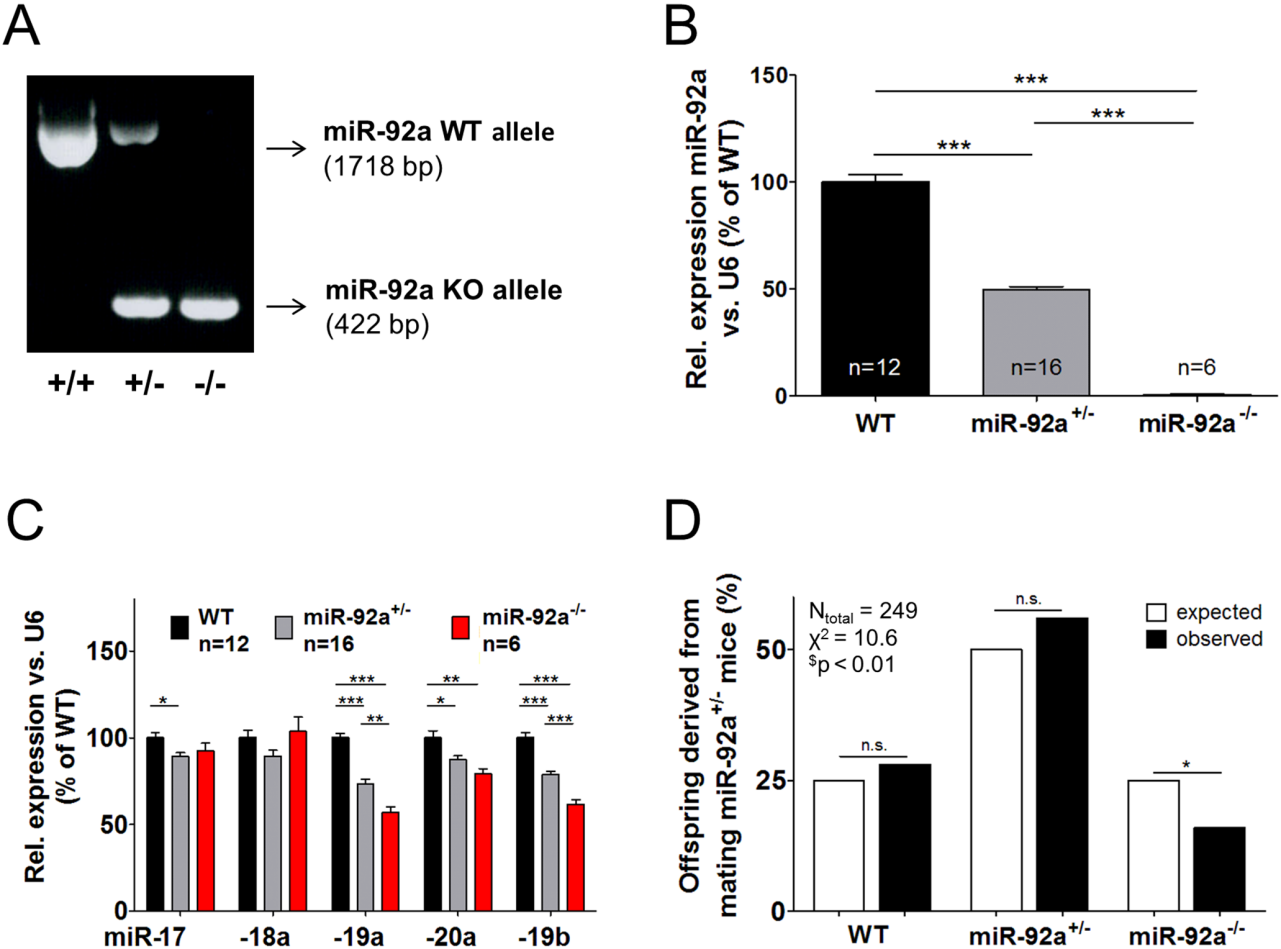
MiR-92a^−/−^ mice survive at a reduced Mendelian ratio. (**A**) Representative agarose gel picture of PCR products (WT allele: 1718 bp; miR-92a knockout (KO) allele: 422 bp) for genotyping of WT, miR-92a^+/−^ and miR-92a^−/−^ mice. (**B**) MiR-92a expression in the heart of WT, miR-92a^+/−^ and miR-92a^−/−^ mice. (**C**) Expression of the miR-17∼92 cluster members miR-17, miR-18a, miR-19a, miR-20a and miR-19b in the heart of WT, miR-92a^+/−^ and miR-92a^−/−^ mice. (**D**) Observed as well as by Mendelian ratios predicted percentage of weaned WT, miR-92a^+/−^ and miR-92a^−/−^ mice derived from mating of miR-92a^+/−^ mice. Data are represented as mean ± SEM, *P<0.05, **P<0.01, ***P<0.001 by one-way ANOVA; ^#^ *P*<0.05 by Clopper-Pearson interval, ^$^ *P*<0.01 by chi-square test.

MiR-92a^−/−^ mice, generated as described above, lack miR-92a expression in various tissues (Figure 1A/B, Figure S1A/B). To determine whether the deletion of miR-92a might have affected the expression of other cluster members, we additionally detected all other mature members of the family and paralog miRNAs that might have been compensatory up-regulated. MiR-92a^−/−^ mice showed a moderate, but significant decrease in miR-19a, miR-19b, and miR-20a in the heart, whereas only miR-19b and miR-20a were significantly decreased in muscle and miR-18a was significantly reduced in skeletal tissue (Figure 1C, Figure S1A/B). All other cluster members and the paralog microRNAs miR-25 and miR-363 were not changed in miR-92a^−/−^ mice (Figure S1C/D).

### MiR-92a^−/−^ mice showed a partial postnatal lethality

MiR-92a^−/−^ mice are viable and fertile, but a reduced Mendelian ratio was observed suggesting that some mice die during embryonic or early neonatal development (Figure 1D). To determine at which time point miR-92a^−/−^ mice are dying, we harvested embryos at E9.5 and E15.5. At both time points, the number of miR-92a^−/−^ mice reflected the expected Mendelian ratio (Figure S2). However, we detected a loss of mice during the first two postnatal days (63% dead pubs in miR-92a^−/−^ (n = 12 of 19) compared to 29% dead pubs in WT controls, (n = 5 of 17); p<0.05). Surviving mice showed no obvious phenotype and exhibit a regular life span (Figure S3A). The morphology of the heart as well as heart rate and blood pressure were normal (Figure S3B/C/D). Capillary density was similar in muscle tissue of miR-92a^−/−^ compared to WT mice (Figure S3E/F). A defect in vascular or heart development was further excluded by demonstrating that mice lacking miR-92a in endothelial cells or cardiomyocytes showed no embryonic or postnatal developmental defect (Figure S3G/H).

### MiR-92a^−/−^ mice have no hematopoietic defects

**Figure 2:**
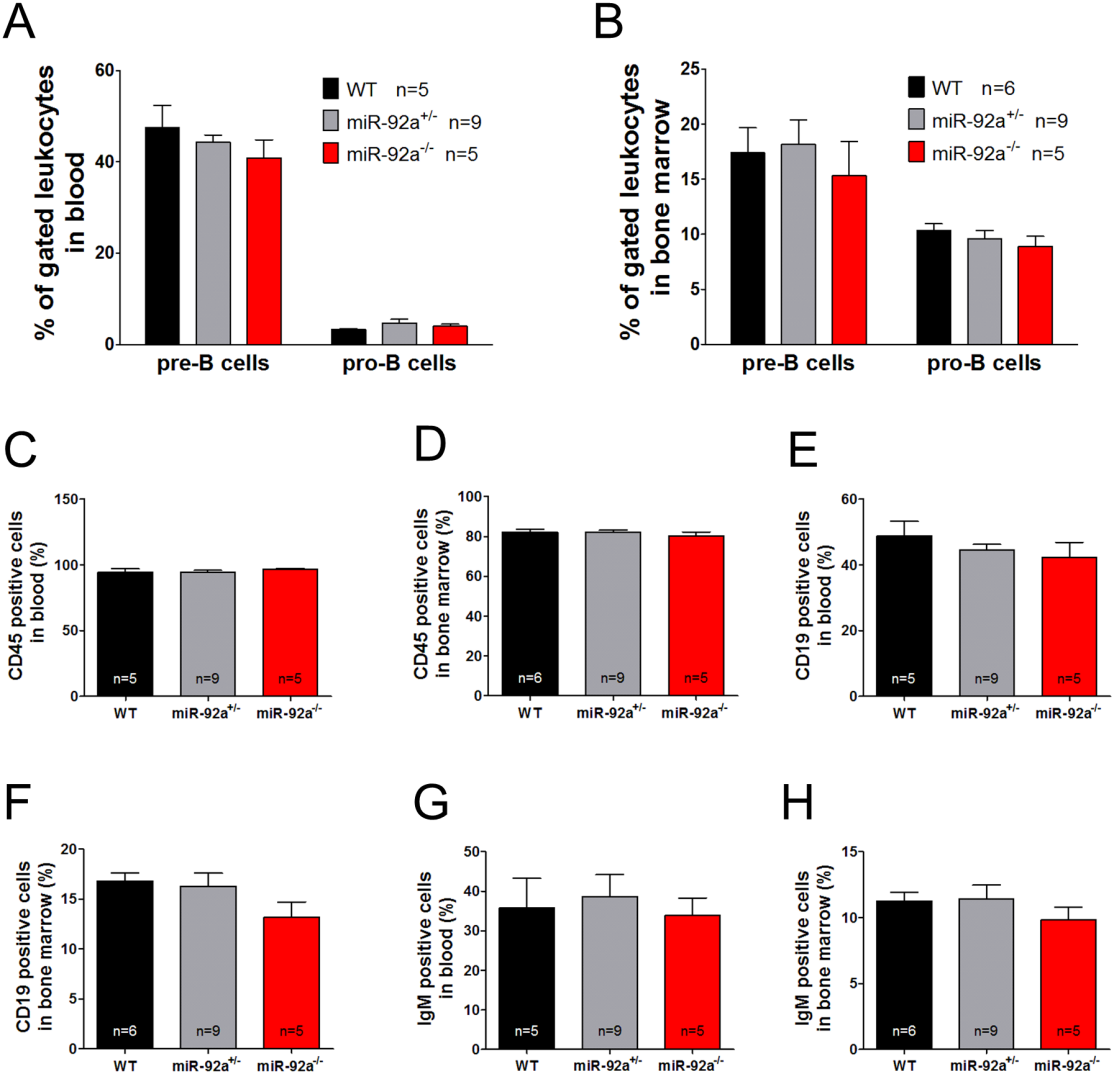
MiR-92a deficiency does not induce obvious defects in the hematopoietic system. Fraction of B220^+^CD43^+^ pro-B cells and B220^+^CD43^−^ pre-B cells measured in blood (**A**) and bone marrow (**B**) of male WT, miR-92a^+/−^ and miR-92a^−/−^ mice. CD45 (**C, D**), CD19 (**E, F**), IgM (**G, H**) positive cells measured in blood and bone marrow of WT, miR-92a^+/−^ and miR-92a^−/−^ mice. Data are represented as mean ± SEM.

Since miR-17∼92 cluster knock-out mice revealed defects in hematopoietic cell development, we characterized the hematopoietic phenotype of miR-92a^−/−^ mice. However, numbers of white blood cells, red blood cells, and platelets, as well as red blood cell parameters were not affected by the knockout (Figure S4A–H). To evaluate a potential effect in B cell development, we additionally determined the number of CD45^+^, CD19^+^, and IgM^+^ cells as well as CD220^+^CD43^−^ pre-B-cells and CD220^+^CD43^+^ pro-B-cells in the peripheral blood and bone marrow. However, none of the cell populations differed in WT versus miR-92a^−/−^ mice (Figure 2). The lack of defects in the hematopoietic system is further supported by the findings that miR-92a^fl/fl^Vav-Cre mice lacking miR-92a in hematopoietic cells do not show any phenotype during embryonic or postnatal development (Figure S4I).

### MiR-92a^−/−^ show growth and skeletal development defect

**Figure 3:**
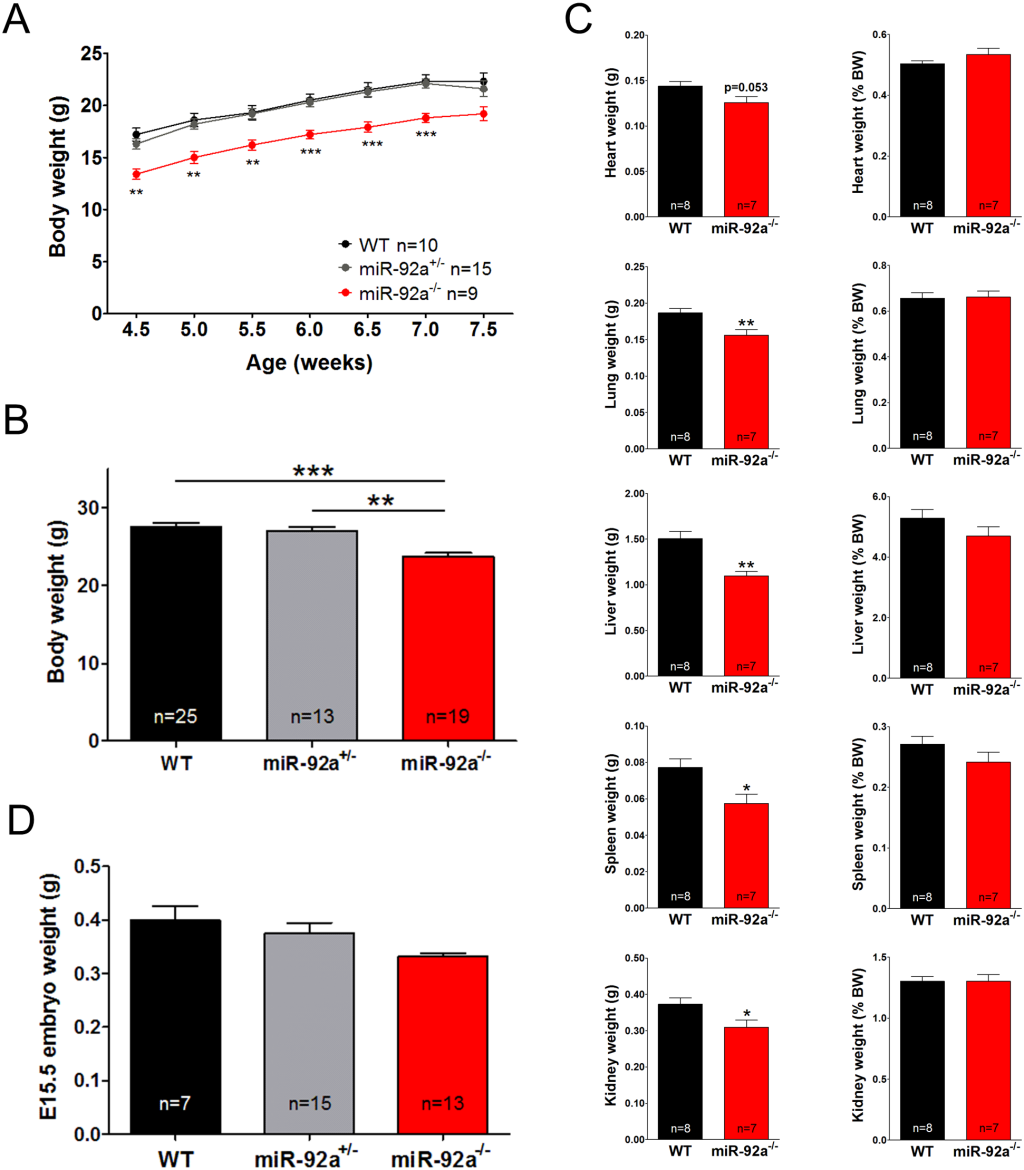
MiR-92a^−/−^ mice exhibit a reduced body weight. Body weight of male juvenile (**A**) and adult (**B**) WT, miR-92a^+/−^ and miR-92a^−/−^ mice. (**C**) Weight of heart, lung, liver, spleen, and kidney of adult male WT, miR-92a^+/−^ and miR-92a^−/−^ mice either as raw data or normalized to body weight. (**D**) Weight of WT, miR-92a^+/−^ and miR-92a^−/−^ E15.5 embryos. Data are represented as mean ± SEM, *P<0.05, **P<0.01, ***P<0.001 by one-way ANOVA in A and B, by student’s t-test in C.

Close examination of miR-92a^−/−^ mice revealed that the body weight was reduced in miR-92a^−/−^ mice during postnatal development and adulthood in both males and females (Figure 3A/B, Figure S5). Interestingly, the weight of all organs was significantly reduced in adult miR-92a^−/−^ mice, however, no significant differences were observed if organ weight was normalized to total body weight suggesting that miR-92a^−/−^ mice are overall simply smaller in size (Figure 3C (right panel)). To determine whether this growth retardation is occurring during embryonic or postnatal development, we further determined the weight of embryos at E15.5. As shown in Figure 3D, miR-92a^−/−^ embryos showed a reduced weight at embryonic day E15.5 indicating that the growth retardation occurs during embryonic development.

**Figure 4:**
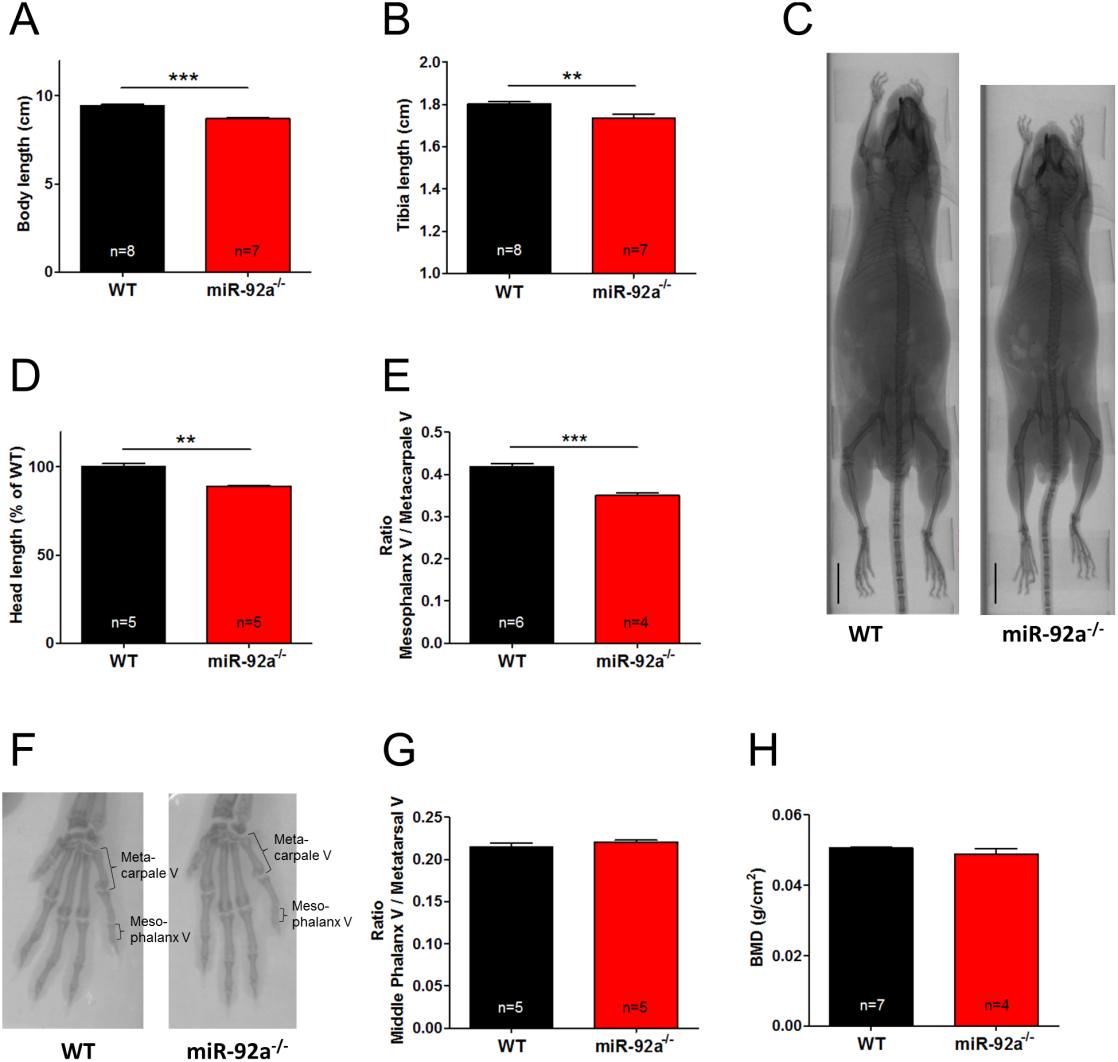
Constitutive deletion of miR-92a in mice causes skeletal defects. (**A**) Body length of WT and miR-92a^−/−^ mice measured from the tip of the nose to the basis of the tail on the animals itself. (**B**) Length of dissected tibias of WT and miR-92a^−/−^ mice. (**C**) Representative pictures of whole body µCT-scans of WT and miR-92a^−/−^ mice, scale bar: 1 cm. Head length (**D**) and ratio of mesophalanx V/metacarpale V of the forelimbs (**E**) measured on µCT-scans of WT and miR-92a^−/−^ mice. (**F**) Representative pictures of µCT-scans of forelimbs from WT and miR-92a^−/−^ mice, mesophalanx V and metacarpale V are indicated. (**G**) Ratio of middle phalanx V/metatarsal V of the hind limbs measured on µCT-scans of WT and miR-92a^−/−^ mice. (**H**) Bone density of WT and miR-92a^−/−^ mice measured by dual energy X-ray absorptiometry. Data are represented as mean ± SEM, **P<0.01, ***P<0.001 by student’s t-test.

To determine whether the reduced size is due to skeletal developmental defects, we quantified body and tibia length. MiR-92a^−/−^ mice showed a significantly reduced full body length as well as tibia length compared to WT littermates (Figure 4A/B/C). In addition, we performed µCT scans and observed that the size of the skull was significantly smaller (Figure 4D). Since hemizygotic deletion of the miR-17∼92 cluster shows specific defects such as brachymesophalangy of the second and fifth fingers, we more closely evaluated the length of the bones in the limbs. Indeed, the length of the 5^th^ mesophalanx to 5^th^ metacarpal bone of the forelimbs was significantly reduced (Figure 4E/F), whereas the ratio of 5^th^ middle phalanx to 5^th^ metatarsal of the hindlimbs was not changed (Figure 4G). To evaluate a potential influence on bone metabolism, we additionally determined bone density. However, the bone density was not different between miR-92a^−/−^ mice and wild type littermates (Figure 4H).

## Discussion

These findings identify a regulatory function for miR-92a in growth and skeletal development, whereas miR-92a is not responsible for other defects in heart or B cell development that were observed in miR-17∼92 cluster mutants. The miR-92a^−/−^ phenotype partially copies the previously reported skeletal development defects of miR-17∼92 cluster knock-out mice [6] and of humans with a reduced expression of the cluster [7]. Thus, miR-92a^−/−^ mice were smaller than their littermates, showed reduced skull size and tibia length and exhibit the typical shortening of the 5^th^ mesophalanx bone as it has been reported for miR-17∼92^Δ/+^ mice [7]. One limitation of the present study, however, is that the deletion of miR-92a moderately affected the expression of miR-20a and miR-19b in heart and muscle tissue, and miR-18a was moderately but significantly reduced in skeletal tissue. Therefore, we cannot fully exclude that the slight reduction in these miRNAs might have contributed to the observed phenotype. However, the reduction of miR-19b and miR-20a in muscle tissue of miR-92a^−/−^ mice was less than 50%. Moreover, the expression of the closely related family members miR-17 (which only differs from miR-20a by 2 nucleotides) and miR-19a (which only differs from miR-19b by one nucleotide) was not significantly changed, and might compensate for the reduction in miR-20a and miR-19b expression, respectively. Moreover, in skeletal tissue only miR-18a was slightly reduced in miR-92a^−/−^ mice.

The mechanism by which the miR-17∼92 cluster affects skeletal development is not well explored. The cluster is highly expressed in bone cells, and osteoblasts from miR-17–92^+/Δ^ mice showed a lower proliferation rate, alkaline phosphatase activity and less calcification *in vitro* [17]. Furthermore, mRNA expression of Runx2 and type I collagen was significantly lower in bones from miR-17–92^+/Δ^ mice [17]. These data may suggest that the miR-17∼92 cluster regulates bone metabolism. However, the present study suggests that miR-92a predominantly contributes to developmental defects but seems not to interfere with bone metabolism. Bone density was not affected in adult miR-92a^−/−^ mice, suggesting that deletion of miR-92a at least under baseline conditions does not significantly affect bone metabolism. Moreover, we did not observe effects of miR-92a inhibition on osteoblast or chondrocytes proliferation or apoptosis in vitro (data not shown). These findings are in agreement with studies showing that pharmacological inhibition of miR-92a does not influence skeletal anabolic responses to mechanical loading [18]. Moreover, pharmacological inhibition of miR-92a even promoted fracture healing [12].

The molecular targets, through which miR-92a regulates skeletal development, are unclear. The miR-17∼92 cluster has been shown to modulate TGF-β signaling, one of the most important signaling pathways controlling skeletal development. However, only miR-17 and miR-20a were shown to directly target TGF-β receptor II and miR-18a was reported to target the TGF-β down-stream signaling proteins Smad2 and Smad4 (for review see [4]). Moreover, the miR-17∼92 cluster indirectly interacts with the Sonic Hedgehog (Shh) axes, a pathway that has been implicated in skeletal development [19], [20]. Whereas several reports showed that Shh stimulates the expression of the cluster [4], [19], [20], an interference of the miR-17∼92 cluster with the Shh pathway is only supported by indirect evidence showing that the tumorigenic effects of the miR-17∼92 cluster is dependent on the loss of the Shh receptor Patched [19]. Therefore, it is unclear whether there is a direct effect of miR-17∼92 loss of function on the activity of the Shh pathway. MiR-92a has several predicted or validated additional targets that were shown to regulate skeletal development and likely a combination of derepressed targets contributes to the observed phenotype. Such targets may include BMP7 and Smad7, which are predicted targets of miR-92a, and p63, which is a validated target of miR-92a [21]. Particularly p63 might be interesting, because mutations in humans cause skeletal syndromes with digit abnormalities [22] and its overexpression in mice induced accelerated ossifications in long bone digits and tail bones [23]. Moreover, inactivation of miR-92a in zebrafish increased noggin3 expression leading to an inhibition of Bmp signaling and abnormal behavior of chondrogenic progenitors during pharyngeal cartilage formation [24].

Beside skeletal defects, miR-92a^−/−^ mice revealed an overall reduction of organ sizes. Analysis of the phenotype of mice overexpressing miR-92a targets revealed that mice overexpressing PTEN also showed a dwarfism [25]. Since PTEN is a known target of miR-92a [10], one may speculate that a de-repression of PTEN during embryonic development may have contributed to the observed organ size reduction of miR-92a^−/−^ mice.

Although miR-92a was shown to profoundly affect endothelial cell functions *in vitro* and *in vivo* [11], [13], we did not observe a change in capillary density in uninjured miR-92a^−/−^ mice. This is consistent with our previous findings showing that pharmacological miR-92a inhibition only increased angiogenesis after induction of ischemia, whereas no change of capillary density occurred in non-ischemic control tissue [11]. This may be explained by the fact that miR-92a is profoundly induced in ischemic tissue and inhibition of miR-92a may preferentially block this response. Second, it is well known that endothelial cells are quiescent and the turn-over is very low in uninjured or unstressed conditions, therefore, an overgrowth of endothelial cells is rarely seen unless the angiogenic or migratory response is activated by injury. Indeed, recent studies confirmed that a genetic deletion of miR-92a in endothelial cells improves re-endothelialization after denudation [26].

In conclusion, the findings of the present study provide insights into the function of miR-92a. MiR-92a so far was mainly been described as an oncogene [2] and was shown to impair angiogenesis and recovery after ischemia [11], [27] and promotes atherosclerosis [13], [15], [16]. The current study additionally documents that miR-92a regulates skeletal development. The mechanism (s) underlying the skeletal development defects of miR-92a^−/−^ mice are unclear and require further investigations.

## Supporting Information

Figure S1 MiR-92a deficiency in mice moderately effects the expression of the other miR-17∼92 cluster members in muscle and skeletal tissue. Expression levels of the miR-17∼92 cluster members in lower leg muscles of the hind limbs **(A)** and femurs **(B)** of WT, miR-92a^+/−^ and miR-92a^−/−^ mice. Expression levels of miR-92a paralog miRNAs miR-25 **(C)** and miR-363 **(D)** in heart of WT and miR-92a^−/−^ mice. Data are represented as mean ± SEM, **P<0.01, ***P<0.001 by student’s t-test.
(TIF)

Figure S2 Proportion of miR-92a^−/−^ embryos is in accordance with the expected Mendelian ratio. Observed as well as by Mendelian ratios predicted percentage of E9.5 **(A)** and E15.5 **(B)** WT, miR-92a^+/−^ and miR-92a^−/−^ embryos derived from mating miR-92a^+/−^ mice.
(TIF)

Figure S3 Under basal conditions, miR-92a^−/−^ mice do not show obvious defects of the cardiovascular system. **(A)** Kaplan-Meier survival curve of female and male WT and miR-92a^−/−^ mice. **(B)** Hematoxylin-eosin stain of longitudinal sections of hearts from adult female WT and miR-92a^−/−^ mice. Heart rate **(C)** and systolic blood pressure **(D)** measured by tail-cuff method in adult female WT and miR-92a^−/−^ mice. Representative pictures **(E)** and quantification **(F)** of the vascularization of the lower leg muscles of the hind limb of adult female WT and miR-92a^−/−^ mice determined as ratio of laminin stained capillaries (white) and isolectin stained capillaries (green). Observed as well as by Mendelian ratios predicted percentage of miR-92a^fl/fl^ and miR-92a^fl/fl^Tie2Cre^+/−^ mice (endothelial cell and progenitor-specific miR-92a deletion) **(G)** and miR-92a^fl/fl^αMHC-Cre^+/−^ mice (cardiomyocyte-specific miR-92a deletion) **(H)** derived from mating miR-92a^fl/fl^ with either miR-92a^fl/fl^Tie2Cre^+/−^ or miR-92a^fl/fl^αMHC-Cre^+/−^ mice. Data are represented as mean ± SEM.
(TIF)

Figure S4 Blood parameters are similar in miR-92a^−/−^ compared to miR-92a^+/−^ and WT mice. **(A–H)** Different blood parameters measured in WT, miR-92a^+/−^ and miR-92a^−/−^ mice are presented. **(A)** White blood cells (WBC), **(B)** red blood cells (RBC), **(C)** platelets (PLT), **(D)** mean corpuscular volume (MCV), **(E)** mean corpuscular hemoglobin (MCH), **(F)** mean corpuscular hemoglobin concentration (MCHC), **(G)** hematocrit (HCT), **(H)** hemoglobin (HGB). **(I)** Observed as well as by Mendelian ratios predicted percentage of miR-92a^fl/fl^ and miR-92a^fl/fl^Vav-Cre^+/−^ mice (miR-92a deletion in hematopoietic cells) derived from mating miR-92a^fl/fl^ with miR-92a^fl/fl^Vav-Cre^+/−^ mice. Data are represented as mean ± SEM.
(TIF)

Figure S5 MiR-92a^−/−^ mice postnatally exhibit a reduced body weight. Body weight of female juvenile **(A)** and adult **(B)** WT, miR-92a^+/−^ and miR-92a^−/−^ mice. Data are represented as mean ± SEM, **P<0.01, ***P<0.001 by one-way ANOVA.
(TIF)
