# Supplementary figures and images for: Phenotypic Characterization of miR-92a−/− Mice Reveals an Important Function of miR-92a in Skeletal Development
Source: PLoS One. 2014 Jun 30;9(6):e101153. doi: 10.1371/journal.pone.0101153 (PMC4076267; doi:10.1371/journal.pone.0101153)

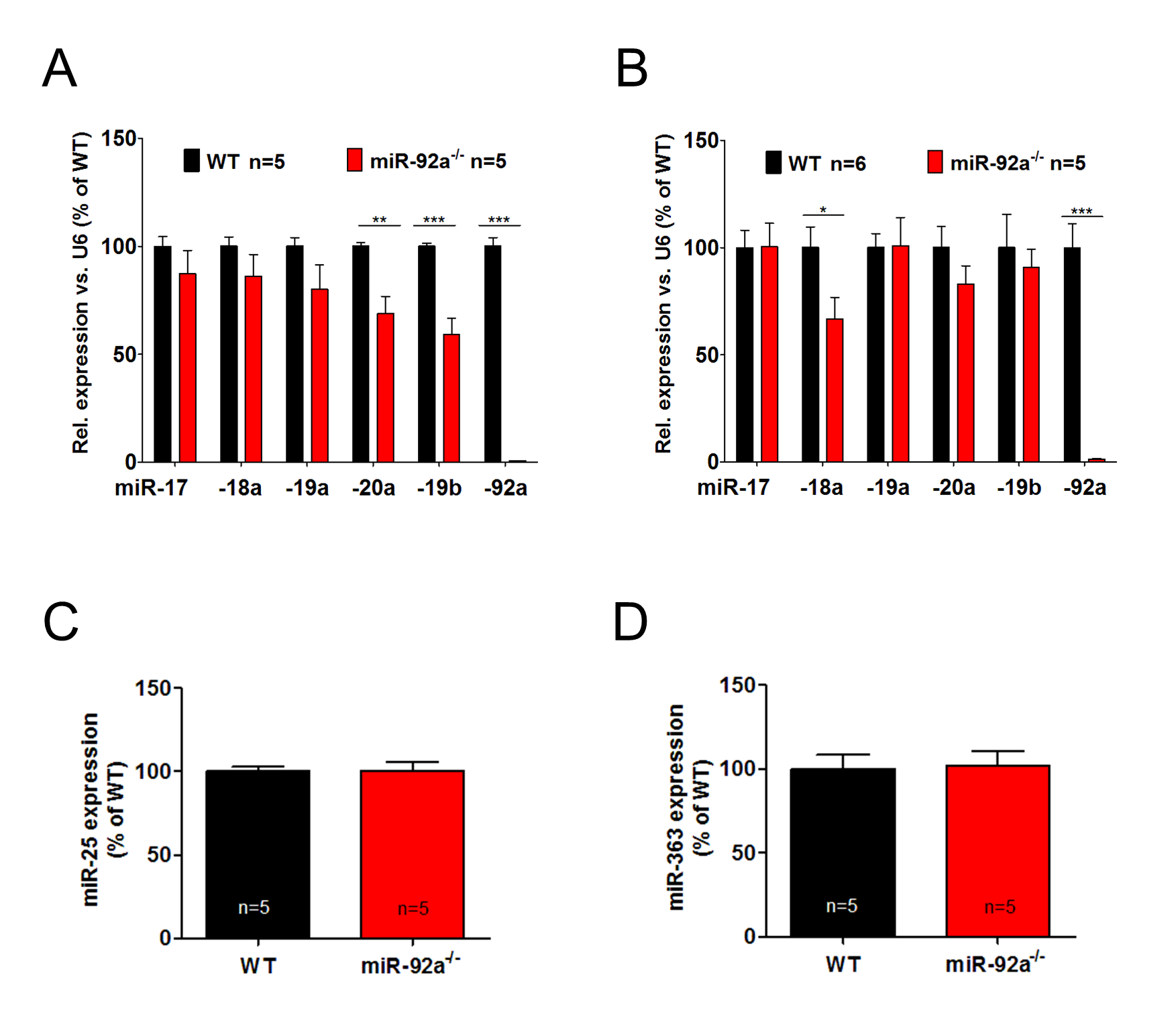

Supplement: Figure S1 — MiR-92a deficiency in mice moderately effects the expression of the other miR-17∼92 cluster members in muscle and skeletal tissue. Expression levels of the miR-17∼92 cluster members in lower leg muscles of the hind limbs (A) and femurs (B) of WT, miR-92a+/− and miR-92a−/− mice. Expression levels of miR-92a paralog miRNAs miR-25 (C) and miR-363 (D) in heart of WT and miR-92a−/− mice. Data are represented as mean ± SEM, **P<0.01, ***P<0.001 by student’s t-test. (TIF) [file pone.0101153.s001.tif]

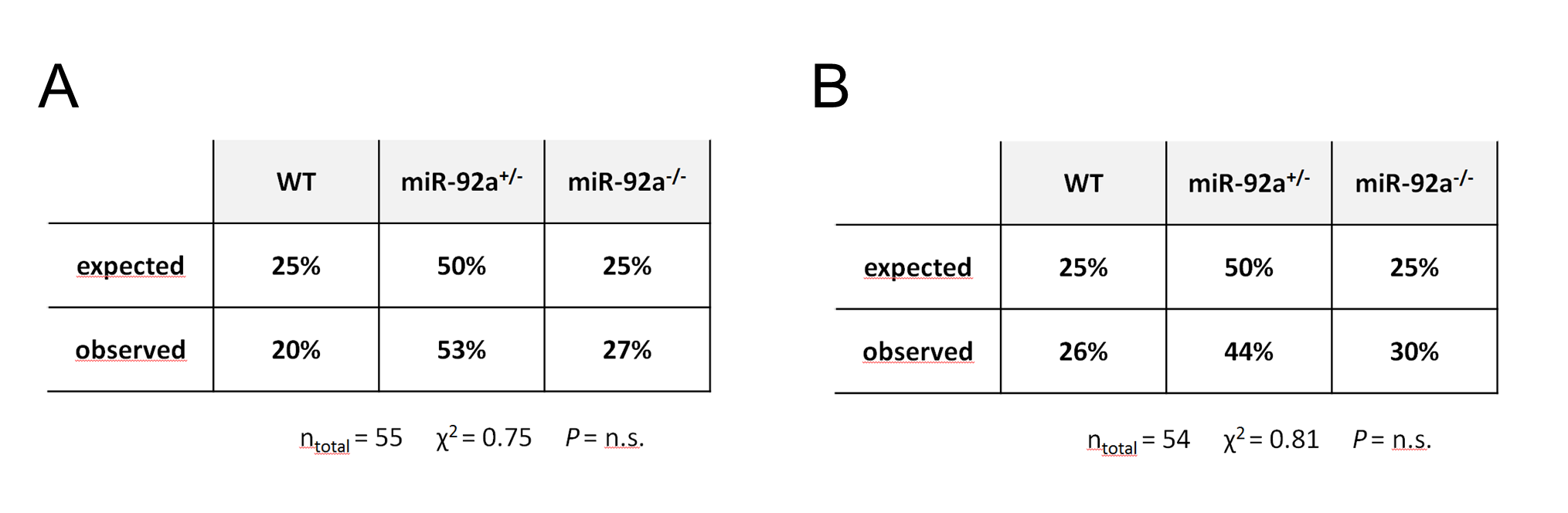

Supplement: Figure S2 — Proportion of miR-92a−/− embryos is in accordance with the expected Mendelian ratio. Observed as well as by Mendelian ratios predicted percentage of E9.5 (A) and E15.5 (B) WT, miR-92a+/− and miR-92a−/− embryos derived from mating miR-92a+/− mice. (TIF) [file pone.0101153.s002.tif]

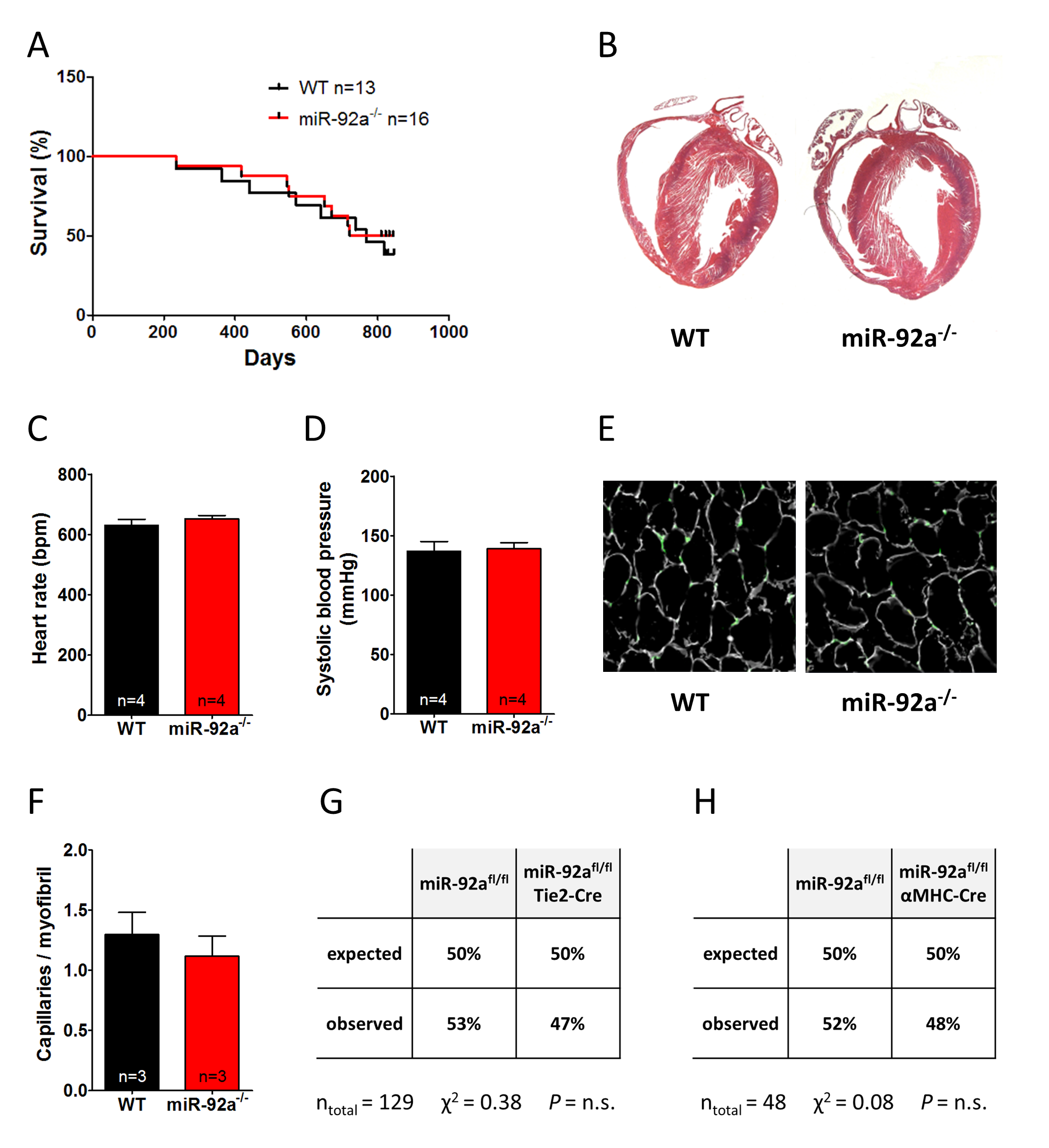

Supplement: Figure S3 — Under basal conditions, miR-92a−/− mice do not show obvious defects of the cardiovascular system. (A) Kaplan-Meier survival curve of female and male WT and miR-92a−/− mice. (B) Hematoxylin-eosin stain of longitudinal sections of hearts from adult female WT and miR-92a−/− mice. Heart rate (C) and systolic blood pressure (D) measured by tail-cuff method in adult female WT and miR-92a−/− mice. Representative pictures (E) and quantification (F) of the vascularization of the lower leg muscles of the hind limb of adult female WT and miR-92a−/− mice determined as ratio of laminin stained capillaries (white) and isolectin stained capillaries (green). Observed as well as by Mendelian ratios predicted percentage of miR-92afl/fl and miR-92afl/flTie2Cre+/− mice (endothelial cell and progenitor-specific miR-92a deletion) (G) and miR-92afl/flαMHC-Cre+/− mice (cardiomyocyte-specific miR-92a deletion) (H) derived from mating miR-92afl/fl with either miR-92afl/flTie2Cre+/− or miR-92afl/flαMHC-Cre+/− mice. Data are represented as mean ± SEM. (TIF) [file pone.0101153.s003.tif]

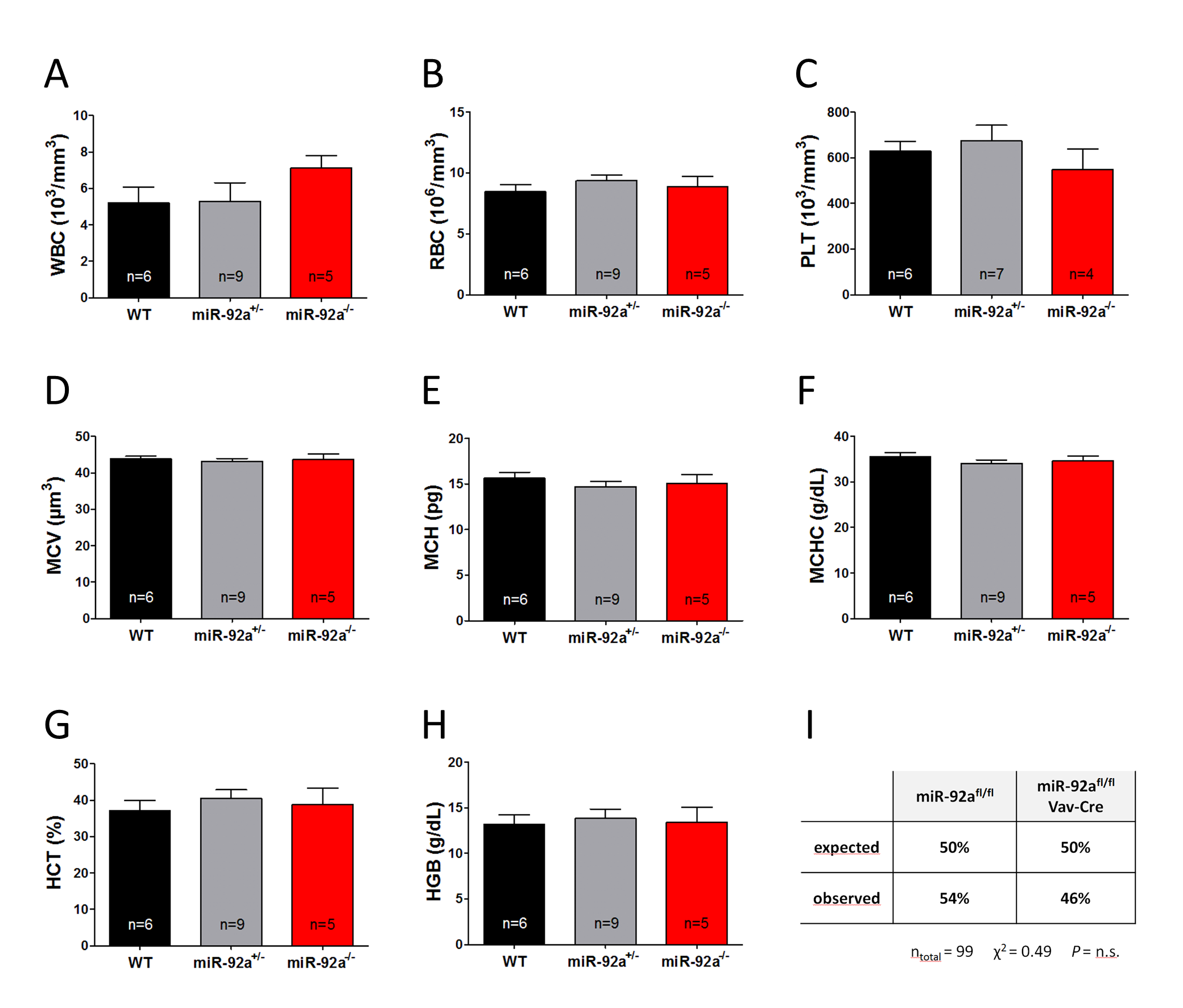

Supplement: Figure S4 — Blood parameters are similar in miR-92a−/− compared to miR-92a+/− and WT mice. (A–H) Different blood parameters measured in WT, miR-92a+/− and miR-92a−/− mice are presented. (A) White blood cells (WBC), (B) red blood cells (RBC), (C) platelets (PLT), (D) mean corpuscular volume (MCV), (E) mean corpuscular hemoglobin (MCH), (F) mean corpuscular hemoglobin concentration (MCHC), (G) hematocrit (HCT), (H) hemoglobin (HGB). (I) Observed as well as by Mendelian ratios predicted percentage of miR-92afl/fl and miR-92afl/flVav-Cre+/− mice (miR-92a deletion in hematopoietic cells) derived from mating miR-92afl/fl with miR-92afl/flVav-Cre+/− mice. Data are represented as mean ± SEM. (TIF) [file pone.0101153.s004.tif]

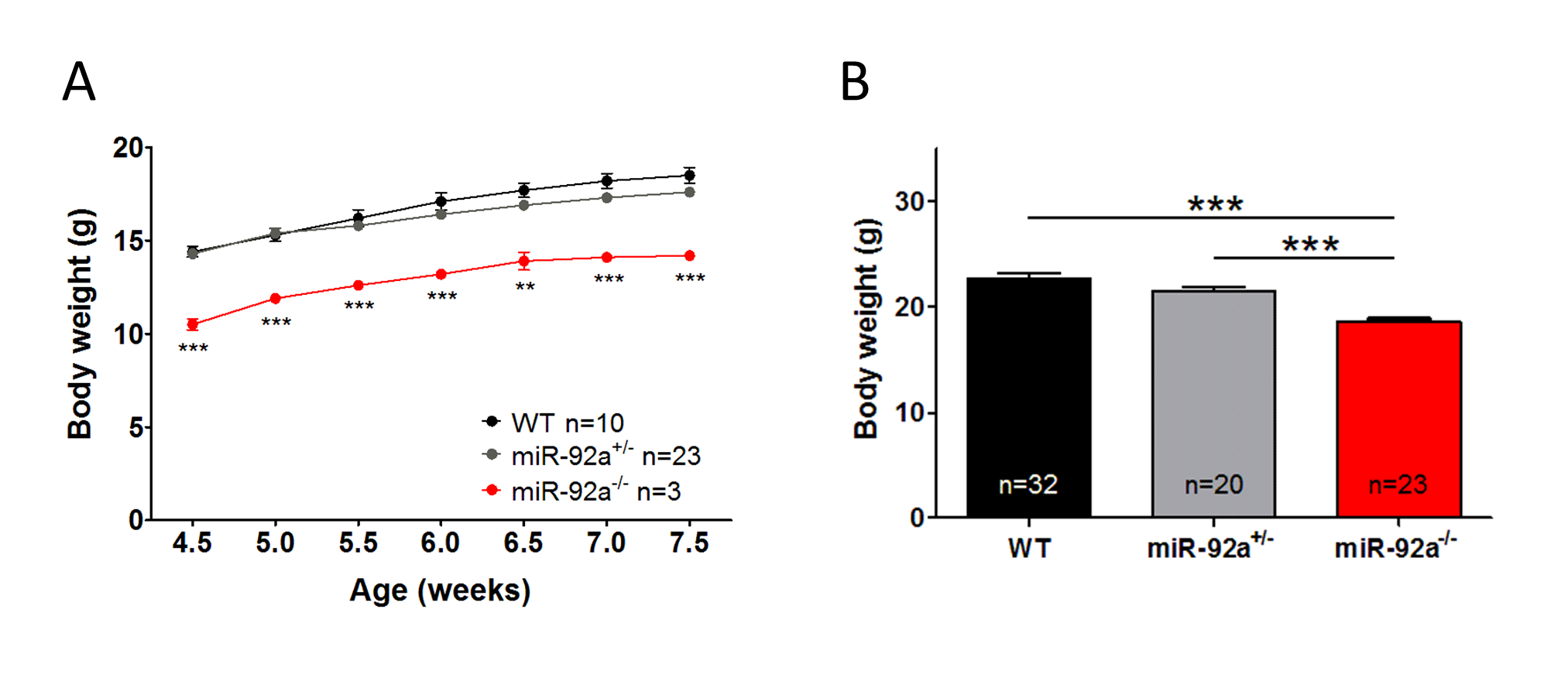

Supplement: Figure S5 — MiR-92a−/− mice postnatally exhibit a reduced body weight. Body weight of female juvenile (A) and adult (B) WT, miR-92a+/− and miR-92a−/− mice. Data are represented as mean ± SEM, **P<0.01, ***P<0.001 by one-way ANOVA. (TIF) [file pone.0101153.s005.tif]
